# Optimising recruitment to a lung cancer screening trial: A comparison of general practitioner and community-based recruitment

**DOI:** 10.1177/09691413231190785

**Published:** 2023-08-01

**Authors:** Hannah Scobie, Kathryn A Robb, Sara Macdonald, Stephen Harrow, Frank Sullivan, in collaboration with the ECLS study team

**Affiliations:** 1 School of Health and Wellbeing, 3526 University of Glasgow, Glasgow, UK; 2 Edinburgh Cancer Centre, Western General Hospital, 3129 NHS Lothian, Edinburgh, UK; 3 School of Medicine, 7486 University of St Andrews, St Andrews, Fife, UK

**Keywords:** Early detection, recruitment, randomised controlled trial, primary care, community, lung cancer, cancer screening

## Abstract

**Objectives:**

Pre-trial focus groups of the Early detection of Cancer of the Lung Scotland (ECLS) trial indicated that those at high risk of lung cancer are more likely to engage with community-based recruitment methods. The current study aimed to understand if general practitioner (GP) and community-based recruitment might attract different groups of people, and to quantitatively explore the demographic and psychosocial differences between people responding to GP or community-based recruitment.

**Design:**

Secondary data analysis of ECLS trial baseline data.

**Methods:**

Adults (n = 11,164) aged 50 to 75 years completed a baseline questionnaire as part of their participation in the ECLS trial. The questionnaire assessed smoking behaviour, health state, health anxiety and illness perception. Alongside demographic characteristics, how participants were made aware of the study/participant recruitment method (GP recruitment/community recruitment) was also obtained via trial records.

**Results:**

The likelihood of being recruited via community-based methods increased as deprivation level decreased. Those recruited via the community had higher levels of perceived personal control of developing lung cancer and were more likely to understand their own risk of developing lung cancer, compared to those who were recruited to the trial via their GP. Health state and health anxiety did not predict recruitment methods in multivariable analysis.

**Conclusions:**

Community and opportunistic screening invitations were associated with uptake in people from less-deprived backgrounds, and therefore might not be the optimal method to reach those at high risk of lung cancer and living in more deprived areas.

## Background

Lung cancer is the leading cause of cancer death worldwide.^1^ Screening for lung cancer offers the best hope for detecting cancers at an earlier and more treatable stage.^2,3^ Targeted lung screening programmes are underway in parts of England^4–6^ and the Early detection of Cancer of the Lung Scotland (ECLS) trial recently reported that those in the intervention arm were detected at an earlier stage compared to those in the control arm.^7^ Lung cancer screening could result in 7000 fewer deaths each year in the UK, but the people most likely to benefit (those experiencing socioeconomic deprivation who have smoked) have historically been the least likely to access cancer screening.^8–10^ The lung screening context therefore provides an important opportunity to challenge the status quo of screening delivery.

The UK Lung Screening Trial reported stark disparities with 19.6% from the lowest (most deprived) Index of Multiple Deprivation (IMD) quintile giving a positive response to a screening invitation, compared with 34.2% in the highest quintile.^11^ A systematic review also found that those with higher socioeconomic status appear to be overrepresented in lung cancer screening programmes, with those from more deprived backgrounds less likely to participate.^12^ The US Preventative Services Task Force (USPSTF) recommendation recognised the urgent need for implementation research to address the most effective ways to increase the uptake of lung cancer screening, particularly in those at higher risk of death from lung cancer.^13^

Lung cancer mortality is highest in the most-deprived groups.^14^ Smokers are disproportionately represented among people living in more deprived areas^15^ and are less likely to participate in screening^10^ making the lung screening population particularly hard to engage. Innovative approaches to increase access are needed or we risk implementing a future lung screening programme that fails to reach those most likely to benefit^16,17^ – a paradox long observed in healthcare, the inverse care law.^18^

There has been limited research exploring the effectiveness of increasing uptake of screening using community-based recruitment. This refers to passive advertisement of screening in local communities where the expectation is that those eligible for screening will ‘self-select’ and contact the relevant healthcare professional. Advertisement of the screening test might include posters, leaflets or stalls. It should be clarified that community-based recruitment does not refer to community-based screening, where screening takes place in the community often using mobile screening units.

This type of community-based recruitment or invitation has not been employed by cancer screening programmes in the UK to date. However, it has been used to recruit participants to cancer screening trials. The ECLS trial used a mix of general practitioner (GP) endorsed invitation letters and community-based recruitment to recruit over 12,000 participants to a randomised controlled trial.^7,19^ Pre-trial focus groups with people at high risk of lung cancer highlighted the acceptability of alternative invitation types, other than (or alongside) a letter from their GP, including word of mouth within the community.^20^ Different forms of media, particularly local newspapers and radio were also seen as a valid form of raising awareness about the study and increasing participation. The insight gained from the focus groups significantly influenced the recruitment method of the lung cancer screening trial. As a result of the feedback, a particular focus was given to community advertisement as a method of recruitment.

The present study aimed to understand if GP and community-based recruitment might attract different groups of people and to quantitatively explore the demographic and psychosocial differences between people responding to these two types of recruitment. The following research questions were addressed:

1. Do people invited to a lung screening trial by GP or community-based recruitment differ in demographic characteristics (socioeconomic status, age, sex, marital status and ethnicity)?
2. Do the beliefs and attitudes towards lung cancer and lung cancer screening differ between people invited to a lung screening trial by GP or community-based recruitment?

## Methods

### Study design

The ECLS trial aimed to develop a new form of lung screening that uses a blood test to identify antibodies that indicate lung cancer.^7^ The trial aimed to recruit 12,000 high-risk participants aged 50 to 75 from deprived areas of Scotland. Participants were adults aged 50 to 75 who were current or former cigarette smokers with at least 20 pack-years or had a history of cigarette smoking less than 20 pack-years plus a family history of lung cancer. The ECLS trial protocol provides details of trial inclusion/exclusion criteria, procedures and questionnaire development.^21^

*GP recruitment*: To recruit participants for the trial, GP practices within the lowest quintile of deprivation measured using the Scottish Index of Multiple Deprivation (SIMD) in NHS Tayside, NHS Greater Glasgow & Clyde and NHS Lanarkshire were approached. A total of 168 practices were approached within these areas, with 166 agreeing to participate in the trial. GP practices were used to help identify eligible patients, and subsequently send out invitations to those identified as eligible. All patients in participating practices were considered with 16% of those eligible being recruited to participate. Potential participants received a letter from their GP practice inviting them to take part in the trial, with those wishing to participate asked to contact the research team directly.

*Community recruitment*: Community recruitment aimed to increase the awareness of the trial and encourage people to make contact if they believed they met the trial inclusion criteria. Community-based advertisement and media campaigns included adverts on TV and radio, posters, flyers, beer mats and other community-based interactions (such as stalls in local hospitals). The majority of community-based recruitment was ‘passive’, with posters and flyers being placed in high traffic areas in the community (e.g. shopping centres and community centres).

All interested individuals identified via community recruitment were assessed in relation to inclusion/exclusion criteria including residence within the selected geographical post codes.

### Measures

*Recruitment method* was established by trial records which indicated if a postal invitation was sent by a GP or not. In the absence of a postal invitation, recruitment was assumed to be via community recruitment. This was cross-checked with the participants’ self-report of recruitment method. In cases (n = 30) where there was a disparity between trial records and self-report, trial record of recruitment method was used.

*Demographic characteristics* were provided by participants before completing the baseline questionnaire (sex, age, marital status and ethnicity). Socioeconomic status was measured using the area-based SIMD.^22^

*Smoking behaviour* was assessed by asking participants whether they had smoked any tobacco products in the past 7 days, with response options: yes or no.

*Health state* was measured using an adapted version of the EQ-5D-3L.^23^ This measure included two subjective measures of health state including a Visual Analogue Health Scale^23^ which required participants to rate their health out of 100 (0 being least healthy and 100 being most healthy). This score was supplemented by the second measure, the Perceived Health State Descriptive Measure.^23^ This five-item measure has been adapted to explore the individual domains of health (mobility, self-care, being able to carry out usual activities, pain/discomfort and anxiety/ depression). Each aspect of perceived health was scored between 1 and 3, for example, 1 = ‘I have no problem walking; 2 = I have some problems in walking about; 3 = I am confined to bed’.

*Health anxiety* was measured using the Health Anxiety subscale of the Health Orientation Scale.^24^ This one-item subscale (‘I feel anxious when I think about my health’) asked participants to rate their level of agreement from 0 (‘not at all’) to 4 (‘very’).

*Illness perception* was measured using an adapted version of the Revised Illness Perception Questionnaire.^25^ This iteration, adapted for the ECLS trial, consisted of seven items. Each component was given a score of 1 to 5. Items were dichotomised into ‘Agree’ and ‘Disagree’. Those who answered, ‘Strongly Disagree’, ‘Disagree’ or ‘Neutral’ were recoded as ‘Disagree’, while those who answered, ‘Strongly Agree’ or ‘Agree’ were recoded as ‘Agree’.

### Analysis

In order to be included within the statistical analyses for this study, participants were required to have taken part in the ECLS trial and completed the baseline study questionnaire. Of the 12,243 ECLS trial participants, 11,164 completed the baseline questionnaire.

Frequencies and means examined the differences between demographic and psychosocial measures of those who responded to GP invitations and those who were reached via community recruitment. Univariate statistical tests included chi-square for categorical variables and Independent Sample T-tests for continuous variables. Univariable and multivariable logistic regression analyses were conducted to examine the associations between demographic and psychosocial factors and cancer screening invitation type. Data analysis was carried out using IBM SPSS V.23.

## Results

### Demographic characteristics of the sample

A total of 11,164 trial participants completed the baseline questionnaire with similar numbers of men (50.6%) and women (49.4%) (see Table 1). Over half of the participants were aged 50 to 60 (53.6%) and were married or in a civil partnership (52.9%). The majority of participants identified as white (99.1%), with only 0.6% identifying with another ethnic group.

**Table 1.** Demographic characteristics by recruitment method, % (n).

	All (n = 11,164)	GP recruitment (n = 9920)	Community recruitment (n = 1943)	Significance
Sex				
*Male*	50.6 (5645)	51.9 (4783)	44.8 (870)	χ^2^(1, 11163) = 32.37, p < 0.001
*Female*	49.4 (5510)	48.1 (4437)	55.2 (1073)	
Age (years)				
*50–60*	53.6 (5982)	53.2 (4906)	55.4 (1076)	χ^2^(2, 11163) = 3.78, p = 0.151
*61–70*	38.7 (4322)	38.9 (3590)	37.7 (732)	
*71–75*	7.7 (859)	7.9 (724)	6.9 (135)	
Marital status				
*Married*	52.9 (5820)	52.2 (4738)	56.2 (1082)	χ^2^(1, 11010) = 10.49, p = 0.001
*Not married*	47.1 (5190)	47.8 (4347)	43.8 (843)	
Ethnicity				
*White*	99.1 (10928)	99.1 (9021)	98.9 (1907)	χ^2^(2, 11030) = 2.31, p = 0.314
*Other ethnic group*	0.6 (69)	0.6 (58)	0.6 (11)	
*Prefer not to say*	0.3 (33)	0.3 (24)	0.5 (9)	
SIMD				
*1 (most deprived)*	40.7 (4534)	43.4 (3994)	28.0 (540)	χ^2^(1, 11130) = 282.41, p < 0.001
*2*	20.1 (2231)	20.5 (1884)	18.0 (347)	
*3*	14.2 (1578)	13.9 (1282)	15.3 (296)	
*4*	14.5 (1614)	13.2 (1217)	20.6 (397)	
*5 (least deprived)*	10.5 (1173)	9.0 (824)	18.1 (349)	

GP: general practitioner; SIMD: Scottish Index of Multiple Deprivation.

The majority of participants were from the most-deprived SIMD groups, groups 1 (40.7%) and 2 (20%). Those from the least-deprived group 5 accounted for 10.5% of participants.

### Univariate analysis

#### Demographic characteristics of GP recruited versus community recruited participants

Women, those who are married, and people from the least-deprived group were significantly more likely to be recruited via the community (Table 1). There was no significant association between age and recruitment method or ethnicity and recruitment method (Table 1).

*Psychosocial measures: Health state –* Participants recruited via the community were more likely to have a higher Health State score (perceived themselves to be healthier), and lower scores in mobility, self-care, usual activities and pain/discomfort, indicating a better health state than those recruited via their GP (Table 2). There was no difference in anxiety/depression scores between those recruited via the community and those recruited via their GP (Table 2).

**Table 2.** Psychosocial measures (health state and anxiety) by recruitment method, mean (SD).

	All (n = 11,164)	GP recruitment (n = 9920)	Community recruitment (n = 1943)	Significance
**Perceived health score (0–100)**	78.7 (17.97)	78.38 (18.13)	80.31 (17.16)	t (10917) = −4.27, p < 0.001
**Health state (1–3)**				
*Mobility*	1.3 (.46)	1.31 (.46)	1.24 (.43)	t (11080) = −5.91, p < 0.001
*Self-care*	1.11 (.32)	1.11 (.33)	1.09 (.29)	t (11049) = −3.37, p = 0.001
*Usual activities*	1.26 (.48)	1.28 (.48)	1.21 (.44)	t (11061) = −5.28, p < 0.001
*Pain/discomfort*	1.54 (.63)	1.55 (.64)	1.47 (.62)	t (11076) = −5.59, p < 0.001
*Anxiety/depression*	1.28 (.51)	1.28 (.52)	1.26 (.49)	t (11062) = −1.78, p = 0.076
**Health anxiety** *‘I feel anxious when I think about my health’*	2.56 (1.19)	2.57 (1.20)	2.50 (1.14)	t (11010) = 2.491, p = 0.013

GP: general practitioner; SD: standard deviation.

*Psychosocial measures: Illness perception –* People recruited from the community reported greater personal control, that is, perception that their actions could control their risk of lung cancer, compared to those recruited via their GP (Table 3). Those recruited via the community were also more impassive about their risk of lung cancer, less unsure about their risk of getting lung cancer, less likely to think lung cancer lasts for a long time, and less likely to think a blood test can detect lung cancer than people who were recruited via GP letter (Table 3).

**Table 3.** Psychosocial measures (illness perception and smoking status) by recruitment method (%).

	All (n = 11,164)	GP recruitment (n = 9920)	Community recruitment (n = 1943)	Significance
**Revised Illness Perception Questionnaire, % agree**				
*Personal control – ‘What I do can affect my risk of lung cancer’*	89.5	89.0	92.2	χ^2^(1, 11029) = 18.15, p < 0.001
*Emotional representation – ‘When I think about my risk of lung cancer, I get upset’*	42.4	43.0	39.6	χ^2^(1, 10992) = 7.61, p = 0.006
*Illness coherence – ‘I don’t know how likely it is that I might get lung cancer’*	64.5	65.2	61.1	χ^2^(1, 10929) = 11.69, p = 0.001
*Treatment control – ‘Finding lung cancer early can improve my chances of survival’*	97.1	97.1	97.2	χ^2^(1, 11008) = .02, p = 0.883
*Consequences – ‘Lung cancer would have a big impact on my life’*	96.1	95.9	96.8	χ^2^(1, 11022) = 3.36, p = 0.067
*Timeline – ‘Lung cancer lasts for a long time’*	64.1	64.7	61.3	χ^2^(1, 10900) = 7.83, p = 0.005
*Treatment control – ‘A blood test can accurately detect lung cancer’*	62.6	64.1	55.4	χ^2^(1, 11014) = 50.99, p < 0.001
**Smoking status, % yes**				
*‘Have you smoked any cigarettes or tobacco in the last 7 days?’*	55.7	58.3	43.7	χ^2^(1, 10130) = 130.55 p < 0.001

GP: general practitioner.

*Psychosocial measures: Smoking status* – People recruited via the community were much less likely to report having smoked in the past 7 days than those recruited by a GP letter (Table 3).

### Multivariate analysis

Multiple logistic regression analysis was conducted to examine how each variable was associated with recruitment source (GP recruitment = 0; community recruitment = 1). Only those variables found to be significant in univariate analyses were included in the multivariate analyses.

The analysis was conducted in two stages to understand the contribution each of the distinct factors made in relation to recruitment source. The first model included demographic variables only and the second model included both demographic variables and psychosocial variables (Table 4). The final model explained 7.6% (Nagelkerke R^2^) of the variance in recruitment source and correctly classified 81.5% of cases.

**Table 4.** Multiple logistic regression (0 = GP recruitment; 1 = community recruitment).

Variable	Model 1 (Demographic characteristics)		Model 2 (Demographic characteristics and psychosocial measures)	
	OR [95% CI]	Significance	OR [95% CI]	Significance
**Sex**				
*Male*	1		1	
*Female*	1.37[1.24, 1.54]	p < 0.001	1.37 [1.24, 1.53]	p < 0.001
**Marital status**				
*Not married*	1		1	
*Married*	1.05 [.94, 1.18]	p = 0.358	1.01 [.90, 1.13]	p = 0.855
**SIMD**				
*1 (most deprived)*	1		1	
*2*	.31[.26, .36]	p < 0.001	.36 [.30, .43]	p < 0.001
*3*	.43 [.36, .52]	p < 0.001	.48 [.40, .58]	p < 0.001
*4*	.53 [.44, .64]	p < 0.001	.56 [.46, .68]	p < 0.001
*5 (least deprived)*	.77 [.64, .92]	p = 0.004	.79 [.66, .95]	p = 0.013
**Health state: Perceived health score**			1.00 [.99, 1.00]	p = 0.868
**Health state: Mobility**			.91 [.76, 1.10]	p = 0.328
**Health state: Self-car**			1.02 [.81, 1.31]	p = 0.853
**Health state: Usual activities**			.96 [.81, 1.16]	p = 0.360
**Health State: Pain/discomfort**			.94 [.83, 1.06]	p = 0.096
**Health anxiety**			1.00 [.95, 1.05]	p = 0.987
**Revised Illness Perception Questionnaire**				
*Personal control – ‘What I do can affect my risk of lung cancer’*				
Agree			1.55 [1.27, .1.89]	p < 0.001
Disagree			1	
*Emotional response – ‘When I think about my risk of lung cancer, I get upset’*				
Agree			.98 [.87, 1.11]	p = 0.747
Disagree			1	
*Illness coherence – ‘I don’t know how likely it is that I might get lung cancer’*				
Agree			.86 [.77, .97]	p = 0.011
Disagree			1	
*Timeline – ‘Lung cancer lasts for a long time’*				
Agree			.94 [.84, 1.06]	p = 0.323
Disagree			1	
*Treatment control – ‘A blood test can accurately detect lung cancer’*				
Agree			.79 [.71, .89]	p < 0.001
Disagree			1	
**Smoking status**				
*‘Have you smoked any cigarettes or tobacco in the last 7 days?’*				
Yes			1	
No			1.72 [1.54, 1.93]	p < 0.001
Nagelkerke *R*^2^	.051		.076	

CI: confidence interval; OR: odds ratio; SIMD: Scottish Index of Multiple Deprivation.

*Demographic characteristics:* Women and people from the least-deprived group had higher odds of being recruited via the community (Table 4). There was no significant difference in marital status across recruitment sources.

*Health state and Health anxiety:* The model indicated that the visual analogue perceived health state score, mobility, self-care, activity, and pain/discomfort did not predict recruitment source (Table 4). Similarly, health anxiety did not significantly contribute to the model in the multivariate analysis (Table 4).

*Illness perception:* Those with greater perceived personal control had higher odds of being recruited via the community. Participants had lower odds of being recruited via the community if they did not know how likely it was that they would get lung cancer and if they considered a blood test could detect lung cancer (Table 4).

*Smoking status:* Those who had not smoked in the last week had higher odds of being recruited via the community (Table 4).

## Discussion

The aim of the current study was to understand if GP and community-based recruitment might attract different types of people and to quantitatively explore the demographic and psychosocial differences between people responding to GP or community-based recruitment. The socioeconomic status of participants did differ significantly between GP-recruited and community-recruited participants: as deprivation level decreased, the likelihood of community-based recruitment increased. In both univariate and multivariate analyses, those from more affluent groups were more likely to self-refer via community-based recruitment. It suggests that passive community-based recruitment is not associated with engaging people from more deprived backgrounds in cancer screening as suggested by the pre-trial qualitative work.^20^

There were some demographic differences seen between the two recruitment types. Of significance, the sex of the participant was a predictor of recruitment type. Univariate and multivariate analysis indicated that women were more likely to be recruited via the community. This is not an unusual finding, with women typically being seen to be more proactive with their health and access to healthcare, compared to men.^26^ This is also entirely in keeping with previous research, which indicates that women are more likely to engage with cancer screening in general,^8,27^ and therefore might be more likely to be more motivated to ‘self-select’ to screen.

Those recruited via community advertisement were significantly less likely to have smoked in the past week. They were also more likely to understand the control they might have over their own risk of developing lung cancer and therefore might have been more likely to self-refer to participate in the screening trial. This might be reflective of the ‘worried well’ phenomenon who undertake screening to confirm their belief that they are either not at risk of lung cancer or for reassurance that they do not currently have cancer.^28^

Those who were recruited via their GP were less likely to understand cancer risk factors and their own risk of developing lung cancer. However, they were more likely to believe that a blood test can accurately detect lung cancer, compared to those recruited via community means. Participants’ beliefs about lung cancer and lung cancer screening also reflect the current literature. Having lower perceived personal control has previously been found to be incompatible with undertaking protective health behaviours.^29^ Those who were recruited via their GP indicated a lack of understanding of their own risk of developing lung cancer and chose to participate prompted by a GP-endorsed letter.^30^ Without such an invitation, and as a result of their low perceived control and personal risk, the GP-recruited group might not have participated in the screening trial.

The results reported indicate that those who self-referred to the ECLS trial via the passive community-based recruitment were more likely to be from more affluent groups. This somewhat contradicts the findings of pre-trial focus groups, which concluded that those who are high risk and from deprived groups might prefer, and better engage with, screening if recruited via alternative community methods such as local TV advertisement and posters placed in high footfall areas (such as GP practices and community centres).^20^ A mix of recruitment methods in the trial was justified, with participants also showing support for GP-based recruitment.^20^ It was important that the ECLS trial organisers acted upon the feedback from the pre-trial focus groups and proactively worked with potential participants to optimise engagement with the trial. This was a positive approach to co-design, although it was recognised at the time that community-based recruitment had not been systematically evaluated.^20^ It does appear that GP-endorsed letters, such as those used in current UK national screening programmes, are the most effective means to invite deprived groups to participate in lung cancer screening.^30,31^ Existing literature indicates that interventions using GP-endorsed letters to increase screening uptake are based on the idea that people trust their healthcare provider to give recommendations, and a letter from them provides a level of personalisation that encourages participation as well as informed choice.^9^ The findings of this study are supported by other studies exploring screening uptake. Analysis of the US-based National Lung Screening Trial (NLST) recruitment methods indicates that uptake is greatest when invitations are received directly by post, compared to passive advertisement and community outreach.^32^ Similarly, the Lung Screen Uptake Trial (LSUT), a UK-based study that used GP letters as invitations to a screening pilot, noted high levels of uptake, with targeted invitations being more effective at engaging more deprived groups.^33^ Although the ECLS trial successfully recruited those living in deprived areas via their GP, more can be done to optimise this type of invitation. For example, evidence indicates that GP endorsement letters should ideally be electronically signed by the GP, and on practice-headed paper, as letters sent on behalf of the practice are generally less effective.^34^

### Strengths and limitations

There were no previous studies that explored the predictors of uptake of a cancer screening trial by recruitment source identified; this study therefore makes a unique contribution to the literature. A strength of this study was that participants were recruited from multiple regions of Scotland, with participation targeted at those who are most at risk of lung cancer. The three study sites used in the trial were selected because of their high levels of deprivation and consequently increased incidence of lung cancer. The study participants were drawn from the most socioeconomically deprived practices in Scotland, which implies that the findings may be generalisable to the people who are at high risk of lung cancer in similar populations. Further research is required to understand the best way to recruit ethnic minority groups to cancer screening trials and improve our understanding of the processes leading to inequalities in cancer screening uptake.

The ECLS trial was a randomised controlled trial to test the effectiveness of a lung cancer screening test. As a result, the trial was not a ‘true’ screening test akin to the national screening programmes in the UK. For example, Clark et al.^35^ highlight the significant issue of representation in clinical trials and indicate the need to improve diversity within clinical trial recruitment. While it is noted that participating in a cancer screening trial is not the same as participating in a screening programme, given that there is no current national lung screening programme, it is useful to draw on the findings of this study to understand the screening behaviour of high-risk groups with the aim to help shape future screening programmes.

There could be ‘study contamination’ across recruitment groups, with people receiving GP letters also potentially being exposed to community-based advertisement. Those from the GP recruitment group could have been influenced by receiving the letter and seeing posters in their community that might further influence participation, compared to those who received only a GP letter or community advertisement. In order to understand the true effectiveness of different recruitment methods, an alternative study design could be implemented. By only using one method of recruitment per area, for example, differences in uptake and demographics of those engaging with each method would be clearer.

## Conclusions

The findings of this analysis are indicative that community recruitment to screening trials attracts higher uptake from more affluent groups, when compared to those who receive an invitation to screening via GP-endorsed letters, such as those used in current national cancer screening programmes in the UK. It is hypothesised that community-based recruitment and other opportunistic screening methods attract those from more affluent communities. As a result, this method of recruitment may further widen cancer screening inequality unless a range of recruitment methods are deployed. Further effort should be given to enhancing GP-based recruitment.
